# Expression of *CSF1*, *AR*, and *SRD5A2* during Postnatal Development of the Boar Reproductive Tract

**DOI:** 10.3390/ani12172167

**Published:** 2022-08-24

**Authors:** Kimberley Katleba, Erin Legacki, Trish Berger

**Affiliations:** Department of Animal Science, University of California, Davis, CA 95616, USA

**Keywords:** 5-alpha reductase type 2, macrophage, leukocyte, estrogen, DHT

## Abstract

**Simple Summary:**

Understanding the initial development of the male reproductive system, including the prostate, should provide insight into malfunctions in the adult male. Although changes in circulating androgens during development are characterized in multiple species, potential changes in the androgen receptor, in the enzyme that converts testosterone to the presumably more potent dihydrotestosterone, and in colony stimulating factor 1, a critical mediator of macrophage influence on organ development, were previously unknown and anticipated to be influenced by androgens and estrogens. Gene expression in the testis, prostate, and seminal vesicles of these three mediators of development, including responses to reduced testosterone or estrogens, were evaluated. Each of these three genes had a unique temporal pattern of expression during postnatal reproductive tract development. However, surprisingly minimal effects of altered steroid signaling were reported on the expression of these presumed pivotal genes.

**Abstract:**

The male reproductive system develops from a minimally functioning gonad and nonfunctioning accessory sex glands in the neonate; sex steroids, presumed to be primary influencers of these changes, have been characterized in multiple species. This study focused on the expression of the androgen receptor as the principal mediator of androgen-induced signaling; the 5α reductase enzyme that converts testosterone to the more active dihydrotestosterone; and colony stimulating factor 1, a mediator of macrophage influence on organ development in the pig. The time points chosen to evaluate normal developmental changes during the juvenile and prepubertal intervals included the inflection time points of 6.5 weeks of age at the nadir of circulating estradiol and testosterone concentrations in juveniles, and 11 weeks of age, when these concentrations begin to increase. The role of sex steroid signaling in the regulation of gene expression was evaluated by the blockade of androgen and estrogen receptors and reduction in endogenous estrogens. Expression of colony stimulating factor 1 in the testes gradually decreased during development; developmental profiles in the prostate and seminal vesicles were clearly different. Interference with sex steroid signaling had no effect on the expression of these three genes in testicular tissue and minimal and transient effects in prostate and seminal vesicles.

## 1. Introduction

Development of the male reproductive system continues from the prenatal interval through the juvenile and pubertal intervals, during which functional competence is gradually acquired. The testes may be considered the foci of this development, but changes in additional organs, including the accessory sex glands, are part of this postnatal development. Laboratory mice complete this process expeditiously, whereas the slightly slower development in pigs allows more distinct separation of these developmental events. Profiles of systemic hormones in the postnatal male pig have been characterized previously [1,2,3,4,5] and include a gradual drop in testosterone and estradiol from birth to 6 weeks of age, an approximate month with low circulating testosterone and estradiol, followed by a gradual increase in circulating levels, which reach a peak at puberty (approximately 4–5 months of age). The pattern of steroid concentrations in testicular tissue resembles circulating levels [6] and may be more significant than circulating concentrations for testicular development. The ability to convert steroids to their more active steroid metabolites (e.g., testosterone to dihydrotestosterone (DHT)) may have local effects on development, particularly in accessory sex glands. Contributions from macrophages and other components of the immune system to organogenesis are now recognized [7,8,9] and may contribute to reproductive tract development as well, but components and their roles have only been only minimally characterized in the postnatal development of the reproductive tract. In other systems, macrophage functions are influenced by estrogen and androgen signaling (reviewed by [10,11]).

Circulating testosterone is converted intracellularly to DHT by 5α-reductase enzymes in peripheral target tissues. In human males, DHT has 2–5 times higher binding affinity than testosterone for the androgen receptor (AR) [12], a 10-fold higher potency for inducing AR signaling than testosterone in the rat prostate [13] and it is essential for the normal development of the prostate, penis, and scrotum in multiple species (reviewed [14]). DHT is the principal active metabolite of testosterone within the boar reproductive tract, with varying amounts present in different tissues [15,16].

The 5α-reductase type 1 isozyme (SRD5A1) has low expression in human embryonic tissues and is expressed at birth in non-genital skin, liver, and certain brain regions, with relatively high expression throughout postnatal human life. Its expression in the epididymis, seminal vesicles, prostate, and genital skin remains low [17]. The 5α-reductase type 2 isozyme (SRD5A2) is expressed in external genital tissues early in gestation. In adulthood, its expression is high in the human epididymis, seminal vesicles, prostate, genital skin, and liver, but remains low in other tissues [18,19]. Presence of 5α-reductase has been detected in the prepubertal and pubertal boar testis [20], and SRD5A2 specifically has been detected in the postpubertal boar epididymis [21]. Both SRD5A1 and SRD5A2 have been identified in the prepubertal and postpubertal boar prostate [22], although type 2 appears to be the dominant isozyme [21].

While androgens are thought to be the major hormone group responsible for male reproductive development, estrogens play a major role in maintaining accessory sex glands postpubertally in the boar [23]. Involvement of estradiol in development of accessory sex glands in juvenile pigs is unknown, although nuclear estrogen receptors (ESR1 and ESR2) are present in the reproductive tract of the young juvenile male pig, as expected from other species (reviewed by [24]). Reducing endogenous estrogens during the juvenile interval in the boar (by inhibiting synthesis from androgen by the aromatase enzyme, CYP19A, with the aromatase inhibitor letrozole) delays testicular maturation [25] and postpubertal accessory sex glands tend to be smaller [26]. Changes in endogenous estrogens might affect macrophages and their effect on tissue development, as is the case in the female reproductive tract [27,28]. However, whether estradiol modulates gene expression and specifically macrophage-mediated signaling in accessory sex glands from juvenile pigs is unknown.

Macrophages, beyond classical immune function, play a role in organogenesis, spermatogenesis, and hormone production. Macrophage presence in the male reproductive tract was previously reported; macrophages may represent one fourth of the cells in the testicular interstitium, although density does vary among species [29]. In seminal vesicles, CD45+ leukocytes, a categorization that includes macrophages, are present in the lumens of the secretory epithelium [30]. Macrophages also appear in the stroma of the seminal vesicles and prostate and are dependent on colony stimulating factor 1 (CSF1) for recruitment, maintenance, and proliferation, as in other tissues [31,32,33]. In the porcine testis, Leydig cells appeared to be the predominant source of CSF1 by immunolabelling and CSF1 presence was also documented in the porcine prostate and seminal vesicles [34]. Consistent with CSF1 presence in Leydig cells, testicular macrophages are intimately associated with Leydig cells and can directly secrete factors that influence steroidogenesis in Leydig cells [35]. In the adult, testicular macrophages play a role in Leydig cell development by providing growth and differentiating factors [36]. Hence, macrophages, CSF1 and steroidogenesis are interconnected in the testis. Presence of SRD5A in human leukocytes [37], with DHT as the main product [38], also connects human macrophages with localized steroid signaling, which is consistent with similar results in murine macrophages [39]. The AR on macrophages are connected with an AR-induced cascade of macrophage recruitment, followed by proliferation of prostate tissue in adult prostatic pathologies [40,41], further suggesting interaction of CSF1, AR and SRD5A2 with each other and with macrophage involvement in normal male reproductive tract development and maturation.

The pig is an ideal species for further evaluation of the contributions of DHT, macrophages, AR and CSF1 to postnatal reproductive tract development in a relatively short developmental time frame. Similarities to humans in genetics, anatomy, and physiology mean such comparisons are particularly valuable. Furthermore, the porcine immune system is well-characterized [42]. The pig is also an excellent animal model to study the effects of estrogens on the mammalian male reproductive tract because the testes produce high levels of circulating estrogens [43,44] and reducing endogenous estrogens does not detectably alter androgens, pituitary gonadotropins, inhibin or prolactin [6,45]. Although the boar has a longer life span (around 1–2 decades) than most laboratory rodent species, puberty occurs at 4–6 months of age [46,47,48]. This time frame allows for the study of specific time points during reproductive tract development.

The present studies were designed to further our understanding of postnatal reproductive tract development (testis, prostate, and seminal vesicles) by evaluating expression of *CSF1*, which contributes to macrophage recruitment and proliferation, and *SRD5A2* and *AR* as potentially important local regulators of this development. Changes in gene expression mediated by androgen and estrogen signaling were anticipated and evaluated using an aromatase inhibitor, letrozole, to reduce endogenous estrogen and an ESR1 and ESR2 antagonist, fulvestrant (also a GPER agonist), to evaluate estrogen signaling, and an AR antagonist, flutamide, to evaluate androgen signaling.

## 2. Materials and Methods

### 2.1. Animals

Pigs shared a common genetic background, originating as breeding stock and semen originating from PIC North America (Hendersonville, TN, USA). Tissues were obtained from 67 animals following euthanasia at the designated ages, flash frozen on dry ice, and stored at −80 °C prior to analysis of gene expression. Additional pieces of tissue were fixed in 4% paraformaldehyde in PBS, dehydrated, and embedded in paraffin for immunolocalization of specific proteins.

Tissues from 32 control animals were used to describe typical gene expression at 2, 6.5, 11, 16, 20 and 40 weeks of age (Table 1). The 2-week age represents an early juvenile age; at 6.5 weeks of age, the neonatally high, systemic testosterone declined to a low level; at 11 weeks of age, systemic testosterone begins to increase, representing a prepubertal transition point; 16 weeks of age represents a pubertal landmark, as a few sperm are typically present in the epididymis, at 20 weeks of age, these animals are sufficiently mature to have enough sperm in the cauda epididymis to inseminate a single female so represent either late puberty or early maturity, and animals at 40 weeks of age represent a more mature state. All control animals were treated with canola oil vehicle weekly from 1 week of age through to 6 weeks of age (except for animals providing tissues recovered at 2 weeks of age). The effect of endogenous estradiol was examined in additional littermates (Table 1) treated weekly with 0.1 mg letrozole (CGS 20267; 4-4′-(1 H-1,2,3-triazol-1-yl-methylene)-bis-benzonitrile; Ciba-Geigy, Basel, Switzerland))/kg body weight, beginning at 1 week of age and ending at 6 weeks of age or 1 week before tissue collection, whichever came first. Six additional animals received daily treatment with fulvestrant, an ESR1 and ESR2 antagonist and GPER agonist (Tocris USA, Ellisville, MO, USA) at a minimum dose of 125 μg/kg body weight through 6.5 weeks of age; these six animals were littermates to six control animals and six animals treated with letrozole and were described previously, including the systemic hormone levels [45]. Eight additional males were treated daily with 10 mg flutamide/kg body weight [49] from 1 to 6 weeks of age, with four animals providing tissues at 6.5 weeks of age and four animals providing tissues at 11 weeks of age. These animals were littermates to control animals and to animals treated with letrozole.

Blood collected from control boars between 2 and 15 weeks of age was stored in PAXgene blood RNA tubes at −20 °C (PreAnalytiX; Qiagen, Germantown, MD, USA; cat # 762165) or Tempus blood RNA tubes stored at 4 °C (ThermoFisher Scientific; Waltham, MA, USA; cat # 4342792) prior to analyzing peripheral blood leukocyte mRNA.

### 2.2. RNA Extraction and cDNA Synthesis

Frozen tissues were immersed in liquid nitrogen and pulverized in a mortar and pestle. Blood RNA tubes were centrifuged at 5000× *g* for 10 min. Total RNA was extracted from both types of samples using Trizol^®^ reagent (1 mL; Invitrogen; Waltham, MA, USA) and total RNA was purified by chloroform extraction (0.2 mL), then incubated at room temperature for 3 min. Samples were centrifuged for 15 min at 12,000× *g* at 4 °C and the aqueous layer was collected and precipitated with 0.5 mL 100% isopropanol, incubated at room temperature for 10 min, then centrifuged for 10 min at 12,000× *g* at 4 °C. The supernate was removed and the pellet was washed with 75% ethanol, vortexed, then centrifuged at 7500× *g* at 4 °C, and the washing procedure repeated. The RNA pellet was air dried and re-suspended in RNAse-free water, then incubated at 55 °C. RNA concentration was determined using a NanoDrop 2000 spectrophotometer (ThermoFisher Scientific), the A260/A280 used to evaluate RNA purity, and RNA stored at −80 °C.

RNA samples were treated with DNAse (M6101; Promega; Madison, WI, USA) to remove genomic DNA. First strand cDNA synthesis was completed with the ThermoFisher Scientific RevertAid first strand cDNA synthesis kit (#K1621). A negative control, containing all reagents except the RNA template, and a positive control using GAPDH (glyceraldehyde-3-phosphate dehydrogenase) were included with each run [50].

### 2.3. qRT-PCR

Real-time quantitative PCR reactions were set up in triplicate using Fast SYBR Green mastermix (#4385612; Applied Biosystems; Waltham, MA, USA) and an Applied Biosystems 7500 Fast Real Time PCR machine. Final reaction volume was 20 µL, containing cDNA synthesized from 5 ng RNA and the amplification temperature program was 95 °C for 20 s, followed by 40 cycles of 95 °C for 3 s and 60 °C for 30 s; after amplification, the melt curve was initiated. Standard curves were generated for all primer pairs (Table 2; 75 nM for *AR* primers and 200 nM for remaining primers) using pooled cDNA and using sequential tenfold dilutions; efficiencies were between 90% and 110%. PCR product sizes were verified by agarose gel electrophoresis following traditional PCR and product purity was determined by qRT-PCR melting temperature. The reference gene used was *SPAG7*, as the RNAseq data showed no change in age within the testis and other studies have shown that some standard reference genes, such as GAPDH and ACTB (actin beta), can change in the testis with age [50,51]. Furthermore, evaluation of mRNA levels in multiple human tissues, including the testis, epididymis, seminal vesicles and prostate, indicated reasonably uniform expression of *SPAG7* across all tissues, except skeletal muscle, cardiac muscle and tongue [52,53]; a pilot analysis indicated that *SPAG7* and *GAPDH* would provide equivalent results as the reference gene for seminal vesicle and prostate tissue from developing boars. The cycle threshold (Ct) for the reference gene (average of the three wells) was subtracted from the average Ct for the gene of interest (Ct gene of interest—Ct reference gene) for each sample to give the delta Ct (ΔCt); the lower the ΔCt, the greater the level of gene expression.

### 2.4. Immunohistochemistry

Tissues were fixed in 4% paraformaldehyde in PBS, processed for paraffin embedding and sectioned for immunohistochemical detection of SRD5A2. Following rehydration, sections were blocked with normal goat serum (Vector Elite ABC Rabbit Kit; Vector Laboratories, Burlingame, CA, USA) and incubated for 2 h at room temperature with 1:250 rabbit polyclonal anti-human SRD5A2 (Santa Cruz, CA, USA; cat # SC-20659). Negative control sections were incubated with normal rabbit IgG. After rinsing, sections were incubated with biotinylated anti-rabbit secondary antibody (Vector Elite ABC Rabbit Kit; cat # PK-6101), followed by incubation with ABC reagent. Labeling was visualized using Nova Red (Vector Laboratories; cat # SK-4800). Slides were examined with brightfield illumination using a 20× objective. A QImaging Micropublisher 3.3 digital camera and QCapture Pro software (QImaging Corporation, Burnaby, BC, Canada) were used to record images. Scoring of SRD5A2 immunolabeling in the prostate (scoring from 1 to 5, with 5 labeling being the darkest; viewer blind to treatment) was analyzed in a minimum of 10 fields (2.43 × 10^6^ μm^2^ per field) from each tissue section. All samples from a litter were immunolabeled for SRD5A2 in the same assay and all ages were represented in each assay to minimize effects of potential variation among the individual assays. Labeling intensity standards were prepared to normalize scoring. The leukocyte marker CD45 was similarly detected with some modifications. Slides were incubated overnight at 4 °C with 1:10 mouse monoclonal IgG2a to pig CD45 (AbD Serotec; cat # MCA1447). Negative control sections were incubated with normal mouse IgG. ABC amplification of the signal from the Vector Elite ABC Mouse Kit (Vector Laboratories; cat # PK-6102) was used and labeling was visualized using an AEC Chromagen (Vector Laboratories; cat # SK-4200). Leukocyte localization was analyzed in a minimum of 5 fields from each tissue section. The macrophage specific marker CD68 was detected with a similar procedure. Slides were incubated overnight at 4 °C with 1:100 goat polyclonal IgG to human CD68 (R&D Systems; cat # AF2040) and negative control sections were incubated with normal goat IgG. Labeling was visualized with the AEC Chromagen (Vector Laboratories; cat # SK-4200) following ABC amplification of the signal (Vector Elite ABC Goat Kit, Vector Laboratories; cat # PK-6105). Macrophage localization was analyzed in a minimum of 5 fields from each tissue section.

### 2.5. Statistical Analysis

The ΔCts from qRT-PCR data for *SRD5A1*, *SRD5A2*. *AR*, and *CSF1* and intensity of IHC labeling data were subjected to analysis of variance (ANOVA) or regression using SAS statistical programs (Proc GLM; SAS Statistical Software, SAS Institute Inc., Cary, NC, USA). Treatment was considered a fixed factor and data from the manipulation of steroid signaling were analyzed separately at each age by ANOVA. Response to age was evaluated using a 1-way factorial with age as a fixed factor and differences in expression among the organs evaluated, with organ and age as fixed factors. Contrasts were used to evaluate differences among ages and organs, although degrees of freedom did not allow for all possible comparisons. Values are presented as least squares means +/− pooled standard error of the mean (SEM). Differences were considered significant when *p* < 0.05.

## 3. Results

### 3.1. Gene Expression and Leukocyte/Protein Localization during Normal Testicular Development

**Figure 1:**
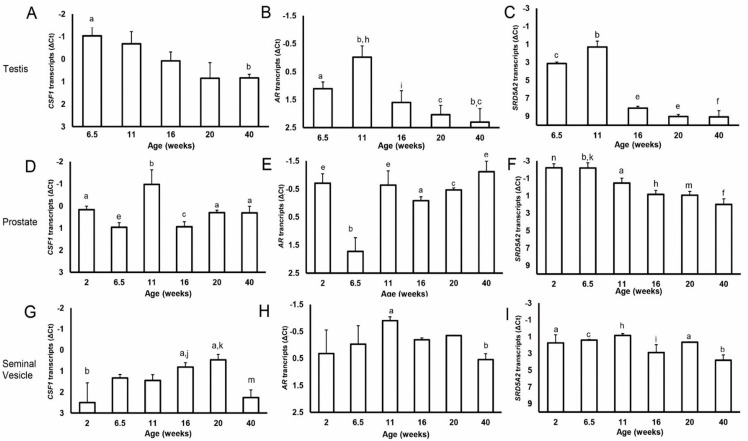
Changes in gene expression (*CSF1*, *AR*, and *SRD5A2*) with age in control boars. (Graphs **A**,**D**,**G**) represent estimates of relative *CSF1* expression in the testis, prostate, and seminal vesicles respectively. (Graphs **B**,**E**,**H**) represent estimates of relative *AR* expression in the testis, prostate, and seminal vesicles, respectively. (Graphs **C**,**F**,**I**) represent estimates of relative *SRD5A2* expression in the testis, prostate and seminal vesicles, respectively. Values represent means and SEM. Columns labeled a,b; h,i; or j,m without common letters differ, *p* < 0.05. Columns labeled b,c; h,n; or k,m without common letters differ, *p* < 0.01. Columns labeled b,e; b,h; c,f; or n,f without common letters differ, *p* < 0.001.

**Figure 2:**
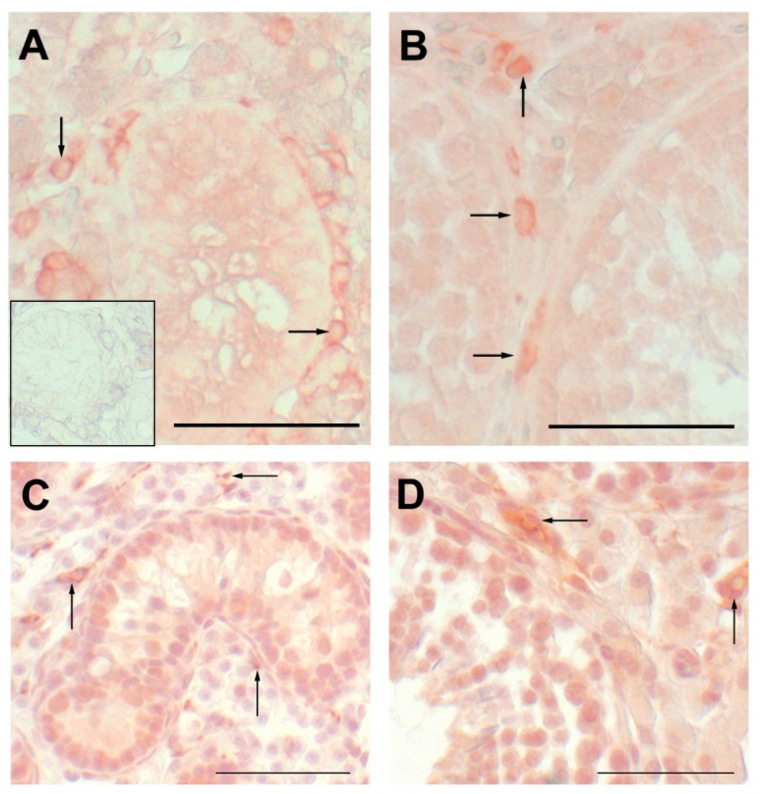
Immunohistochemical labeling of leukocytes (CD45 positive in **A**,**B**) and specifically for macrophages (CD68 positive in **C**,**D**) in the testis. Arrows point to individual leukocytes in testis from a 6.5-week-old boar in (**A**), from a 20-week-old boar in (**B**) and to macrophages in hematoxylin counterstained testis from 6.5-week-old boar in C and from a 20-week-old boar in D. Inset is a normal serum control and is similar to the negatives for the other treatment and age. Scale bar is 50 µm.

**Figure 3:**
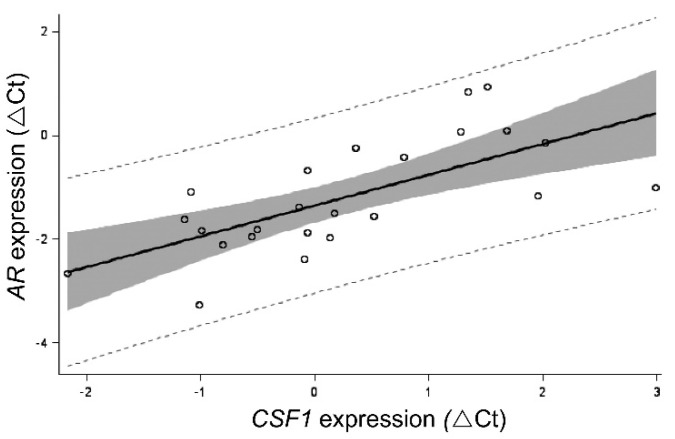
Relationship between *CSF1* expression and *AR* expression in testes from control boars.

**Figure 4:**
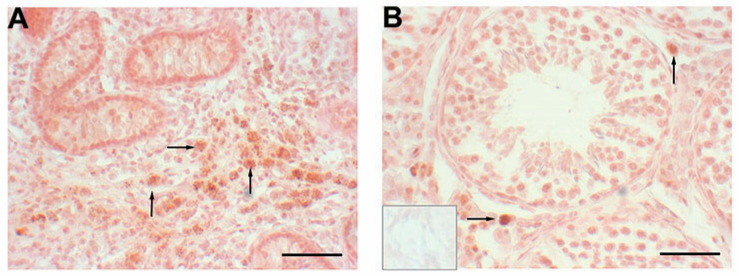
Localization of SRD5A2 protein in testes from a 6.5-week-old boar (**A**) or a 20-week-old boar (**B**). Arrows point to some of the labeled cells. Inset is a normal serum control and is similar to the serum control at 6.5 weeks of age. Scale bar is 50 µm.

Expression of *CSF1* mRNA decreased from the prepubertal through the postpubertal time points within the testis (6.5 vs. 40 weeks, *p* < 0.05; Figure 1). Leukocytes (including macrophages) appear early within the testis (Figure 2). At 6.5 weeks of age, leukocytes are already in close proximity to the seminiferous tubules, where they remain at 20 weeks of age. Testicular expression of *AR* mRNA was affected by age, being highest at the prepubertal transitional time point (11 weeks) (11 vs. 20 weeks, *p* < 0.01; 11 vs. 16 weeks *p* < 0.05; 6.5 vs. 11 weeks and 6.5 vs. 40 weeks *p* < 0.05; Figure 1). Testicular expression of *AR* was positively correlated with *CSF1* expression in vehicle-treated animals (*p* < 0.0005; R = 0.69; Figure 3). Testicular expression of *SRD5A2* mRNA was also affected by age (Figure 1). Overall, expression of *SRD5A2* mRNA is relatively low in the testis, but highest prepubertally at the prepubertal transition point (11 vs. 16 weeks, 11 vs. 20 weeks, 6.5 vs. 40 weeks, *p* < 0.0001; Figure 1). Testicular expression of *CSF1* and *SRD5A2* mRNAs was also positively and significantly correlated (R = 0.55, *p* < 0.05). Expression of *SRD5A2* mRNA and *AR* mRNA within the testis was positively correlated as well (R = 0.67; *p* < 0.0005). Expression of SRD5A2 protein was observed in a small, disperse population of cells within the testicular interstitium at the prepubertal and transitional time points and detected in visibly fewer cells at the pubertal time point (Figure 4).

### 3.2. Gene Expression and Leukocyte/Protein Localization during Normal Prostate Development

**Figure 5:**
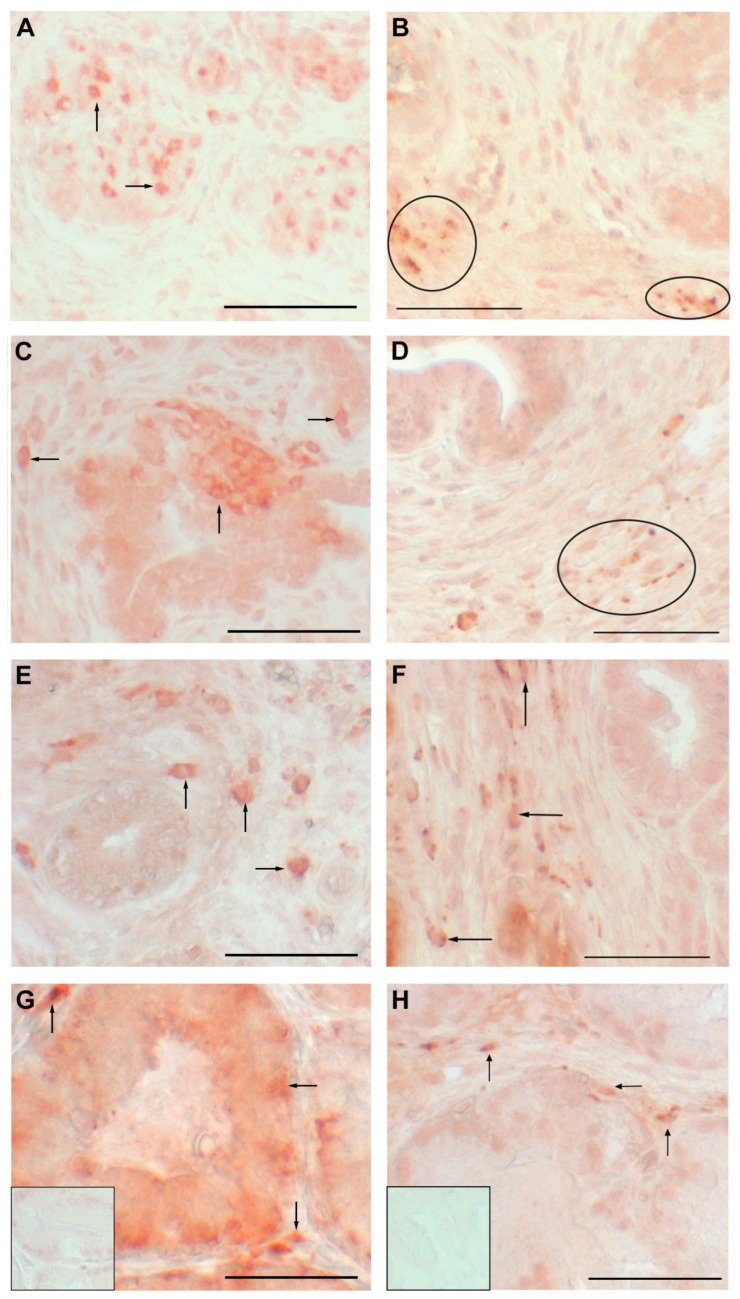
Immunohistochemical localization of leukocytes (**A**,**C**,**E**,**G**) and specifically macrophages (**B**,**D**,**F**,**H**) in the prostate during development. (**A**,**B**) are sections from prostates from 2-week-old boars; (**C**,**D**) are prostate sections from 6.5-week-old boars, (**E**,**F**) are sections from 11-week-old boars; (**G**) is a prostate section from a 40-week-old boar and (**H**) is a prostate section from a 16-week-old boar. Arrows point to individual cells and ovals surround clusters of immunolabeled macrophages. Insets are normal serum controls and similar to the serum controls at other ages. Scale bar is 50 µm.

**Figure 6:**
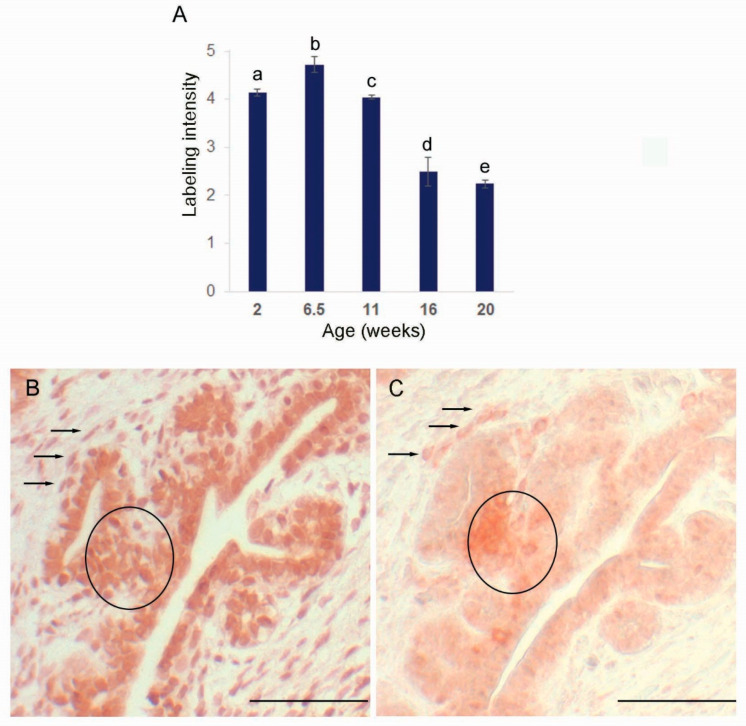
SRD5A2 protein in the porcine prostate. (**A**). Relative intensity of protein labeling in the prostate epithelium. Values represent means and SEM. Columns labeled a,b or b,c differ, *p* < 0.05. Columns labeled a,e; c,d; or c,e differ, *p* < 0.001. (**B**,**C**). Serial sections of prostate from a 6.5-week-old boar immunolabeled for SRD5A2 and for CD45, respectively. Circles and arrows point to a cluster of cells and individual cells, indicating that a minor portion of SRD5A2 expression colocalizes with leukocytes. Scale bar represents 50 µm.

Expression of *CSF1* in the prostate differed from the testicular profile, with an increase at the prepubertal transition point (6.5 vs. 11 weeks, *p* < 0.001; Figure 1) followed by a decrease through the pubertal and postpubertal time points. Leukocytes (including macrophages) are located as clusters in the prostate stroma prepubertally (Figure 5), but appear to be more dispersed during the transition to puberty. At this transition, an increased number of macrophages appear to be present, particularly along the connective tissue and in close proximity to blood vessels. By 16 weeks of age and in more mature animals, leukocytes, including macrophages, are in close proximity to the epithelium (and within the epithelium) and are no longer clustered, but rather appear as scattered single cells. Expression of *AR* mRNA in the prostate was low at 6.5 weeks of age (2 vs. 6.5 weeks and 6.5 weeks vs. 11 weeks, *p* < 0.001; Figure 1). In contrast, prostatic expression of *SRD5A2* mRNA decreased significantly from prepubertal through postpubertal time points (2 vs. 40 weeks, *p* < 0.001; Figure 1). This reduction was most pronounced between the juvenile to prepubertal time points (6.5 vs. 11 weeks, *p* < 0.05). The SRD5A2 protein was highly expressed in prostates from prepubertal boars, consistent with local synthesis and was less prominent in older boars (Figure 6A). Labeling intensity was highest at 6.5 weeks of age for both the epithelium (2 vs. 6.5 weeks *p* < 0.05 and 6.5 vs. 11 weeks, *p* < 0.001; Figure 6A) and stroma (2 vs. 6.5 weeks, *p* < 0.05 and 6.5 vs. 11 weeks, *p* < 0.05), with the labeling intensity being higher in the epithelium than the stroma at the early time points. Typically, protein expression had the same age profile as gene expression.

### 3.3. Gene Expression during Normal Development of the Seminal Vesicles

Expression of *CSF1* mRNA in the seminal vesicles decreased postpubertally (20 vs. 40 weeks *p* < 0.01; Figure 1), was also low at the youngest age examined (2 weeks), and was generally low compared with the testis and prostate (*p* < 0.001). *AR* expression was relatively high in the seminal vesicles compared with the testis (*p* < 0.001) but variable, with a postpubertal decrease (11 vs. 40 weeks, *p* < 0.05). Expression of *SRD5A2* mRNA in the seminal vesicles was relatively low compared with the prostate (*p* < 0.001), but higher than in the testis (*p* < 0.001), and highest at the prepubertal and transitional time points (11 vs. 16 weeks and 6.5 vs. 40 weeks, *p* < 0.05 and *p* < 0.01, respectively), with a small increase at 20 weeks of age (20 vs. 40 weeks, *p* < 0.05).

### 3.4. SRD5A Expression in Other Tissues

Peripheral blood leukocytes demonstrated detectable but low expression of *SRD5A2* from 2 to 15 weeks of age compared with the prostate (*p* < 0.05), with no effect of age (ΔCt of 11.2 ± 0.3), although *AR* was not detected in these peripheral leukocytes during this interval. Leukocytes, including macrophages, may make a small contribution to overall SRD5A2 expression, as partial co-localization of SRD5A2 immunolabeling with the macrophage marker CD68 was observed in serial sections at 6.5 weeks of age (Figure 6B,C). Co-localization can be observed along the tubular structure (branch elongation) or within the stroma (duct formation). Relatively high expression of *SRD5A1* was detectable in the corpus epididymis at 6.5 and 40 weeks of age (ΔCt of −1.03 + 0.36), with reasonably consistent relative expression throughout the epididymis at 6.5 weeks of age (ΔCt of −1.88 and −1.47 in caput and cauda, respectively). However, detectable *SRD5A1* expression was not present in the testis, prostate, or seminal vesicles.

### 3.5. Gene Expression in the Testis following Reduced Estrogenic or Androgenic Signaling

**Figure 7:**
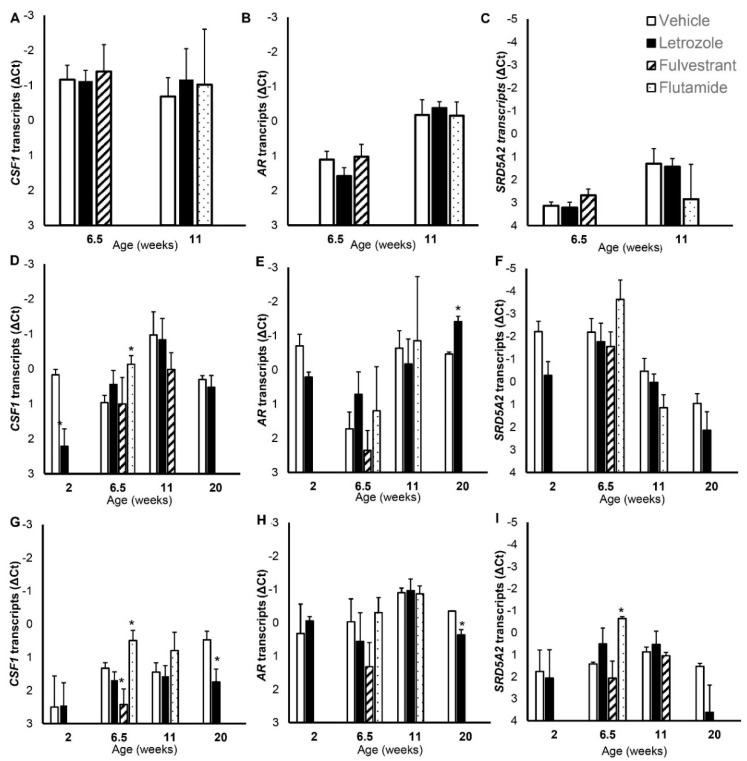
Effects of estrogen and androgen signaling on *CSF1*, *AR*, and *SRD5A2* gene expression in testes (**A**–**C**), prostate (**D**–**F**) and seminal vesicles (**G**–**I**). Values represent means and SEM. Estrogen signaling was reduced in littermates by inhibiting estradiol synthesis with letrozole or by blocking signaling via nuclear estrogen receptors with fulvestrant. Androgen signaling was blocked with flutamide. * indicates mean differs from vehicle-treated littermates, *p* < 0.05.

Reduced endogenous estrogen signaling, by blocking nuclear estrogen receptors with fulvestrant or by inhibiting estrogen synthesis with the aromatase inhibitor letrozole, did not affect testicular *CSF1*, *AR* or *SRD5A2* mRNA expression at the juvenile time point (6.5 weeks of age; Figure 7). Similarly, reducing steroid signaling (estrogen or androgen) did not affect testicular expression of *CSF1*, *AR* or *SRD5A2* mRNA at the prepubertal transition (11 weeks of age).

### 3.6. Gene Expression in the Prostate following Reduced Estrogenic or Androgenic Signaling

Reduction in circulating estrogens (letrozole treatment) decreased *CSF1* mRNA in the neonatal prostate (2 weeks of age) compared with vehicle-treated boars (*p* < 0.05; Figure 7), but the response did not persist during continuously reduced endogenous estradiol nor in response to the estrogen receptor blockade through 6.5 weeks of age. An increase in prostate *CSF1* expression was observed at 6.5 weeks of age following continuous androgen receptor blockade with flutamide, but this response again did not persist past 11 weeks of age. Expression of prostate *AR* was generally nonresponsive to reduced estrogen or androgen signaling, although a small increase in *AR* expression was noted at 20 weeks of age (*p* < 0.05; Figure 7). Interference with steroid signaling did not affect prostate *SRD5A2* at any time point, although reduced estradiol tended to reduce *SRD5A2* expression at 2 weeks of age (*p* = 0.08).

### 3.7. Gene Expression in the Seminal Vesicles Following Reduced Estrogenic or Androgenic Signaling

Expression of seminal vesicle *CSF1* was altered by steroid signaling at 6.5 weeks of age. Blocking the androgen receptor with flutamide increased expression and blocking the estrogen receptor with fulvestrant reduced expression (*p* < 0.05; Figure 7). Reducing estradiol numerically reduced, but did not significantly alter, expression at 6.5 weeks of age. The *CSF1* response to reduced androgen signaling or estrogen receptor blockade did not persist past 11 weeks of age, although *CSF1* expression was reduced at 20 weeks of age following continuously reduced estradiol. Expression of *AR* in the seminal vesicles was generally unresponsive to alterations in steroid signaling, although reduced estrogen signaling (letrozole treatment) decreased *AR* mRNA compared with vehicle-treated littermates at 20 weeks of age (*p* < 0.05; Figure 7). Reduced steroid signaling had minimal effect on *SRD5A2* mRNA expression within the seminal vesicles, with higher expression detected at 6.5-weeks of age in boars treated with flutamide to block androgen receptor activation, compared with littermates treated with the vehicle (*p* < 0.05). However, this response was transient, as no effect was observed at 11 weeks of age.

## 4. Discussion

Macrophages appear early in testis development and seem to be the major leukocyte in the current study, as well as in previous reports [54,55]. Macrophages interact with Leydig cells, influence testosterone production [56,57], and may support spermatogenesis (reviewed by [58]. In the current study, the three genes (*CSF1*, *AR*, and *SRD5A2*) were chosen for expression analysis based on their perceived role in the development of the male reproductive tract and potential interconnection.

Tissue CSF1 is believed to attract macrophages, consequently stimulating growth and development in many organs [7,8,9]. Leydig cells, peritubular myoid cells and macrophages all produce CSF1 within the testis [59,60]. Location of tissue resident macrophages correspond to these CSF1 sources, with some macrophages being intimately associated with Leydig cells and other resident macrophages associated with the peritubular myoid cells surrounding the seminiferous tubules [54,55,61,62,63].

The relatively high expression of *CSF1* in the juvenile testis and decreasing expression with age observed in this study are consistent with the contributory role of CSF1 in testicular development and maturation, as established in other organ systems [64,65,66,67,68]. Expression of testicular *AR* is highest at the prepubertal transition point, a time when circulating testosterone is low; increased expression of AR might be a compensatory mechanism. Expression of *SRD5A2* is quite low in the testis, although the pattern of expression resembles that of *AR* and is obviously different from that of *CSF1*. However, the expression profile is consistent with the changes in testicular SRD5A2 enzymatic activity with age; higher enzymatic activity was present at 6 weeks of age compared with pubertal boars [20]. This previous work also indicated SRD5A activity was present in the microsomal fraction and not the nuclear fraction, consistent with immunohistochemical localization of the SRD5A protein in the cytoplasm observed in the current study.

*CSF1* expression in the prostate was highest at the transition point prior to allometric growth of the prostate. Expression of *CSF1* was perhaps important to stimulate the apparent influx of macrophages at this time point. In contrast to the tissue resident macrophages present in some other organs, the macrophages in the prostate of CSF1-deficient mice were not affected by increased circulating CSF1 [31]. This suggests the need for local *CSF1* production in the prostate, as is known for a few other tissues. Continued expression of *CSF1* at all other time points in the prostate is consistent with this positive local role; however, the role of macrophages in prostate development is unclear. *AR* expression in the prostate is comparatively low at 6.5 weeks of age, at the same time point that circulating testosterone is quite low. At other time points, prostate expression of *AR* is substantial and relatively constant. In contrast to both *AR* and *CSF1* expression profiles, expression of *SRD5A2* in the prostate decreases relatively steadily during development. The SRD5A2 protein was detected immunohistochemically in both the prostate stroma and epithelium, with protein expression appearing higher at the younger ages, which is similar to the higher gene expression during early development. The SRD5A2 in the prostate is key to the local conversion of testosterone to DHT, a particularly potent androgen involved in the maintenance of prostate function. Local activity of the SRD5A2 enzyme, coupled with local conversion of testosterone sulfate to testosterone [69,70,71], suggests local androgenic signaling independent of fluctuations in peripheral testosterone. The importance of SRD5A2 to prostatic development in humans and laboratory rodents is demonstrated by the small and undeveloped prostates present when a mutation in the gene prevents expression of a functional enzyme [14,72]. Previous reports of *SRD5A2* expression in leukocytes [37,38] was confirmed for peripheral leukocytes from young boars. The idea that local leukocytes might contribute to SRD5A2 synthesis in the prostate was demonstrated immunohistochemically; however, this contribution is minor based upon the low expression in leukocytes compared with prostate tissue. A second gene with 5α reductase activity is *SRD5A1*, which is present in the human prostate [17,73,74]. We were unable to detect expression of *SRD5A1* mRNA in porcine prostates, although Palin and colleagues [22] hypothesized the presence of SRD5A1 in the boar prostate based upon a second peak of enzymatic activity.

Expression of *CSF1* in porcine seminal vesicles was low in the neonate and the mature male compared with the testes and prostate and exhibited a unique expression profile compared with these tissues. Androgen receptor expression was high, with the highest expression at the prepubertal transition with the expression profile that was also unique in the seminal vesicles, compared with testes and prostate. Expression of *SRD5A2* in the seminal vesicles was generally lower than that observed in the prostate and with a less consistent pattern of expression during development. Although precise comparisons of small changes in relative gene expression among multiple organs by qRT-PCR would require uniform expression of the selected reference gene among those organs, the differences among organs observed in this study were large.

Overall, each organ displayed a unique expression pattern of these three genes during development. In addition, expression profiles for each of the three genes generally differed within an organ but this study did not evaluate expression profiles by cell type within an organ. Since all three organs exhibit allometric growth during approximately the same ages, this variation among organs is surprising. Furthermore, since seminal vesicles and prostate are both accessory sex glands, the lack of similarity is even more surprising. Gene expression is not necessarily mirrored by subsequent protein expression, as additional regulators of translation exist; this regulation may explain some of the observed differences in gene expression.

Reduced steroid signaling had remarkably minimal effects on the expression of these three genes involved in development. No effects of reduced estrogen signaling were observed on the expression in the testes, although reduced estradiol leads to increased Sertoli cell numbers [25,45]. Reduced androgen signaling similarly had no effect on gene expression in the testes, as was the case with a treatment that affected Sertoli cell numbers [49]. This suggests that testicular expression of these genes in not regulated by estrogenic or androgenic signaling. Reduced estrogen or androgen signaling induced transient reductions in prostatic *CSF1* expression; neither of which persisted beyond a single time point. A small increase in prostatic *AR* expression was similarly detected at a single time point (20 weeks) following reduced testicular estradiol synthesis and lowered circulating estradiol. Although *SRD5A2* is particularly associated with the prostate, with gene expression being relatively high, neither reduced estrogenic nor reduced androgenic signaling affected prostate *SRD5A2* expression. This was surprising and suggests that expression is robust. Thus, the relatively steady decrease in prostate *SRD5A2* expression during development is apparently not regulated by estrogenic or androgenic signaling. Expression of each of these developmental genes in the seminal vesicles was transiently affected by one or more reductions in steroid signaling. However, the data are again more consistent with minor and transient responses to altered steroid signaling, with alterations in such steroid signaling being neutralized with time rather than persisting. The inconsistency between reduced estrogen signaling from reduced estradiol synthesis and from blockading nuclear estrogen receptors also contributes to the perception that steroid regulation of these genes in the seminal vesicles is limited. The current observations of the minimal effects of sex steroid signaling on gene expression during development differ from the anticipated effects of androgen signaling on accessory sex glands. Reductions in androgen signaling increased *AR* expression in adult rat prostate and seminal vesicles [75,76] in contrast to these observations in juvenile pigs, although no effect on testis *AR* was noted in rats, which is similar to the current observations. This is a surprising result and might be due to the differences in the method used to reduce AR signaling, to differences in the juvenile and adult animals, or to species differences. Expression of these perceived pivotal genes were anticipated to change following reduced estrogen signaling, since estrogens were demonstrated to maintain accessory sex glands in adult males following castration [23].

## 5. Conclusions

Although testes, prostate, and seminal vesicles all undergo hypoallometric growth or isometric growth followed by positive allometric growth during development, the expression profiles of *CSF1*, *AR*, and *SRD5A2* are generally unique in each organ. The prostate has relatively high expression of *SRD5A2* prepubertally, which decreases with age, while the testis and seminal vesicles have low expression prepubertally. In the testis, *AR* and *CSF1* mRNA are positively correlated, suggesting that AR may be involved with the recruitment, maintenance, or proliferation of macrophages during testicular development. Furthermore, estrogenic and androgenic regulation appear to be relatively minor contributors to the regulation of these three developmental genes.

## Figures and Tables

**Table 1:** Number of littermate pigs providing specified tissue at each age ^1^.

Age (Treatment)	Testis	Prostate	Seminal Vesicles
2 weeks (control)		4	4
6.5 weeks (control)	9	6	5
11 weeks (control)	5	6	6
16 weeks (control)	4	3	3
20 weeks (control)	3	3	3
40 weeks (control)	3	4	6
2 weeks (letrozole)		4	4
6.5 weeks (letrozole)	9	4	4
11 weeks (letrozole)	5	5	5
20 weeks (letrozole)		3	3
6.5 weeks (fulvestrant)	6	4	4
6.5 weeks (flutamide)		4	3
11 weeks (flutamide)	4	4	4

^1^ All comparisons of responses to altered hormone signaling with control animals utilized only littermates to the specific treated animals.

**Table 2:** Primers used in qPCR analysis of gene expression.

Gene	Gene ID	Forward Primer	Reverse Primer	Source	Size
*SPAG7^49^*	NM_001243921.1	GAGCTGGATTCCTACCGTCG	CCTTCAGCCTCCGTTTCTCC	Millipore Sigma	70
*SRD5A1*	XM_003134156.6	TCTGCACCTACAACGGCTAC	CCTGCTAGAAATCGGGGGTC	Integrated DNA Technologies	94
*SRD5A2*	NM_213988.1	ATGGATCGGCTATGCCTTGG	AGGGCTTTTCGAGACTTGGG	Invitrogen	147
*AR*	NM_214314.2	TACCTGTGTGCCAGCAGAAAT	AGCTCCCAGTGTCATCCCT	Invitrogen	108
*CSF1*	NM_001244523.1	GGTGTCGGAGAACTGTAGCC	ACACTGGATCCGTCAACTGC	Integrated DNA Technologies	137

## Data Availability

Data are contained within the article.
